# Interferon-gamma (IFNy) in macrophage activation syndrome (MAS) associated with systemic juvenile idiopathic arthritis (sJIA). High levels in patients and a role in a murine mas model

**DOI:** 10.1186/1546-0096-12-S1-O3

**Published:** 2014-09-17

**Authors:** Claudia Bracaglia, Ivan Caiello, Kathy De Graaf, Giovanni D'Ario, Florence Guilhot, Walter Ferlin, Lidia Melli, Giusi Prencipe, Sergio Davì, Grant Schulert, Angelo Ravelli, Alexei Grom, Cristina De Min, Fabrizio De Benedetti

**Affiliations:** 1 Division of Rheumatology, Department of Pediatric Medicine, IRCCS Ospedale Pediatrico Bambino Gesù, Rome, Italy; 2 Novimmmune SA, Plan-les-Ouates, Geneva, Switzerland; 3 University of Genoa, Istituto Giannina Gaslini, Genoa, Italy; 4 Division of Pediatric Rheumatology, Cincinnati Children’s Hospital Medical Center, University of Cincinnati College of Medicine, Cincinnati, Ohio, USA

## Introduction

IFNγ is the pivotal mediator in murine models of primary HLH.

## Objectives

Given the similarities between primary and secondary (sHLH), including MAS, we analyzed IFNγ levels in patients with sJIA and MAS and evaluated the pathogenic role of IFNγ in a murine MAS model.

## Methods

We measured levels of IFNγ, IFNγ-related chemokines (CXCL9, CXCL10, CXCL11), and IL-6 in patients with sHLH (n=14), and in patients with sJIA (n=54) of whom 20 had MAS at sampling using the Luminex multiplexing assay and evaluated their relation to disease activity. The effect of the anti-IFNγ antibody XMG1.2 was assessed in IL-6 transgenic (IL6TG) mice in which a MAS-like syndrome leading to death is triggered by TLR ligands (Strippoli, Arthritis Rheum 2012). An LPS LD50 (5 μg/gr body weight) was used, as a trigger for MAS, followed 10 hours later by administration of 100 μg/gr of XMG1.2.

## Results

Levels of IFNγ and of IFNγ-related chemokines [median pg/ml(IQR)] were markedly elevated in active MAS and active sHLH, with no significant differences between active sHLH [IFNγ 34.7(23.9-170.1); CXCL9 33598(3083-127687); CXCL10 4420(799.7-8226); CXCL11 1327(189-2000)] and active MAS [IFNγ 15.4(5.1-52.6); CXCL9 13392(2163-35452); CXCL10 1612(424.8-4309); CXCL11 564.8(197.5-1007)]. Levels in active sJIA without MAS at sampling [IFNγ 4.88(3.2-8.7); CXCL9 836.5(470.9-2505); CXCL10 307.3(198.9-693.7); CXCL11 121.7(62-197.1)] were lower (all p-values <0.01) than in active sHLH or active MAS. IL-6 was not different between the three groups. In active MAS, platelet count was inversely related to IFNγ (r=-0.53; p=0.02), CXCL9 (r=-0.51; p=0.03) and CXCL10 (r=-0.58; p=0.009). In the murine MAS model, treatment with the anti-IFNγ antibody XMG1.2 resulted in increased survival (XMG1.2-treated 10 survivors/10 treated; control-treated 5/10; p=0.033).

## Conclusion

IFNγ, and IFNγ-related chemokine levels were increased in patients with MAS compared to patients with active sJIA without MAS, and associated with low platelet count. Neutralization of IFNγ increased survival in murine MAS.

## Disclosure of interest

None declared.

